# The COVID-19 pandemic as an existential threat: Evidence on young people’s psychological vulnerability using a Multifaceted Threat Scale

**DOI:** 10.1371/journal.pone.0292894

**Published:** 2023-10-12

**Authors:** Mattia Vacchiano, Emanuele Politi, Adrian Lueders

**Affiliations:** 1 Department of Sociology, University of Geneva, Geneva, Switzerland; 2 Laboratory of Social Psychology, University of Lausanne, Lausanne, Switzerland; 3 Centre for Social and Cultural Psychology, KU Leuven, Leuven, Belgium; 4 Institute of Communication Science, University of Hohenheim, Stuttgart, Germany; University of Huelva: Universidad de Huelva, SPAIN

## Abstract

Research offers evidence that younger generations suffered the most psychologically from the COVID-19 crisis. In this article, we look at the onset of the pandemic to understand the reasons for this increased vulnerability. We use the COVID-19 Multifaceted Threat Scale to explore potential mechanisms underlying generational differences in psychological well-being. In a sample of 994 individuals (+18) obtained in the USA and India, we first assess levels of perceived psychological well-being across the generations. Thus, we measure cross-generational differences in the perceived levels of financial, relational, existential, health and lifestyle threats experienced by respondents seven months after the pandemic broke out. In accordance with earlier findings, the results confirm that people from Generation Z and Generation Y reported worse levels of psychological well-being than older adults. Our results suggest that the heightened existential threat, as reflected in a loss of meaning and feelings of being “trapped”, mediate the association between younger generations and worse psychological well-being. No substantial intergenerational differences were found for other threat dimensions. The observed effects were consistent across both national contexts, hence stressing the importance of existential concerns as a mechanism underlying the psychological vulnerability of younger people in the historical contingencies of the COVID-19 pandemic.

## 1. Introduction

Three years on, a picture is emerging of who suffered most psychologically from the outbreak of the COVID-19 pandemic [1]. In the first months of 2020, it became clear that those most at risk from its impact were the cohort of the elderly and those with concurrent pathologies [2]. However, the direct physical health consequences of the pandemic were far from being the only concern that weighed on the population. The evidence accumulated down these years shows that many sensitive factors for psychological well-being emerged from the upheavals to the worlds of work and everyday life [3]. This was not only due to the contingencies of the pandemic as such, but also to the authorities’ attempts to flatten the epidemiological curve and avoid the spread of the virus. That is, many stressors for psychological well-being have arisen from the implementation of lockdowns and the wide range of restrictions on leisure and face-to face encounters [4, 5].

The disruptions caused by the pandemic have therefore had significant repercussions on the well-being of the population. This was markedly different among social groups, with variations observed in different contexts, and especially with an increase in risk factors among people with a vulnerability profile in relation to their health, economic or social status. For example, women, migrants, the economically disadvantaged and people with pre-existing mental health conditions experienced an increase in risk factors during this challenging period [6–11].

Data suggest that negative psychological effects of the pandemic were heightened among the younger generations [12]. Young people have a known susceptibility to mental health problems under normal circumstances, and emerging evidence points to a deterioration in their psychological well-being over the course of the pandemic [13, 14]. In different socio-economic contexts it has been observed that younger generations, such as “Generation X” (born between 1966 and 1980), “Generation Y” (born between 1981 and 1994) or “Generation Z” (born between 1995 and 2012), have shown less resilience in coping with the stresses caused by the pandemic than older generations, e.g., “baby boomers” (between 1946 and 1965), who were most vulnerable to the health consequences of the infection [10, 12, 15].

These generational inequalities can be explained by the fact that older generations, despite being more physically vulnerable, have been least directly affected by the upheavals in the world of work and changes in lifestyle. Older generations tend to live in more stable conditions and are economically more secure than younger generations, for whom the pandemic has caused a range of financial, professional and personal uncertainties [12]. Not only has the pandemic added insecurity to their professional paths, it has also forced many young parents to reorganize family routines and childcare [6, 16]. Lockdown measures have deprived younger generations of leisure and in-person interactions, giving new power to computers and smartphones to maintain social bonds among their peers. Emerging from all this is a “new normal” [17] permeated with sensitive factors for psychological well-being that are linked to threats of all kinds: to one’s health, to one’s life, to one’s finances, to the quality of one’s relationships and to the fulfillment of one’s standard of living [18, 19].

The pandemic thus brought to light an unprecedented range of threats and stressors [20]. Many have been overwhelmed by the unexpected economic uncertainties and the growing concern about whether living standards could be maintained at pre-pandemic levels. They feel at risk of running out of money, not being able to meet their basic financial obligations and seeing their way of life altered, limiting travel and leisure activities [21]. In the context of such a traumatic global event, deeper existential threats have emerged, giving rise to feelings of purposelessness and a diminished sense of life. Isolation led many to feel being trapped, questioning the meaning of their daily routines. The struggle to find purpose in this crisis has thus become a deep concern for many [18, 22]. Relational threats have emerged due to the need to move away physically and have limited social interactions. People long for the company of friends and family, making the absence of social contact tangible. Loneliness and nostalgia for normal social interactions have become pregnant issues [12]. Health threats such as the fear of an unknown disease and inadequate treatment emerged early on, in a context of great uncertainty about the possibility of curing the virus [23].

In sum, the pandemic has unleashed a complex web of concerns. While the key focus of the pandemic´s management focused on the prevention of dangers to physical health, the detrimental psychological outcomes of the pandemic are still revealing themselves. The present article aims to explore the impact of the COVID-19 pandemic on psychological well-being across different generations and to describe the psychological mechanisms that can help explain potential intergenerational differences. At the heart of the project lies a comparison of different manifestations of threat that are sensitive to the specific burdens that each generation had to carry during the course of the pandemic [18, 22]. With this aim in view, the COVID-19 Multifaceted Threat Scale employed here distinguished between perceived health, financial, relational, lifestyle and existential threats. This psychometric tool was constructed to measure a comprehensive range of threats experienced during the virus outbreak using an inductive and bottom-up approach to item generation. The full process of constructing of this psychometric tool is explained in [18].

Using this psychometric tool, we test hypotheses in two different socioeconomic and cultural contexts, namely the United States and India, two countries that were among the hardest hit by the proliferation of the virus in September 2020 [2]. First, in accordance with prior evidence, we expect significant intergenerational differences in well-being. More precisely, we expected younger cohorts to report lower levels of well-being as compared to older adults (H1). Once this hypothesis is tested and verified, we look for potential explanatory mechanisms through the COVID-19 Multifaceted Threat Scale. We thus expected to see significant variation across age cohorts in the experience of pandemic-induced threats (H2). We do not formulate a priori hypotheses about the exact manifestation of these proposed variations within the specific domains that are part of the threat scale. As a third hypothesis (H3), we expected intergenerational variations in such threat reports to explain the proposed differences in well-being.

## 2. Materials and methods

### 2.1. Participants

#### 2.1.1. Country-specific sample characteristics

The data used in this study were obtained through the Anonymized project (full-length title) conducted by [*Anonymous Information*]. The project aimed to examine psychological well-being during the COVID-19 pandemic and involved administering a survey to participants who had provided written informed consent. The research was approved by the ethical committee of the Australian Catholic University (2020-113E; 2020-114E). The data was anonymized to ensure confidentiality. The research was conducted in two different countries, namely the United States and India, chosen for their substantial socioeconomic and cultural differences [24–27]. This selection facilitated the exploration of cross-cultural variations in the perception of threat within the global context of the COVID-19 pandemic and allowed for hypotheses about psychological health to be tested not only in the frequently studied Western countries [28, 29]. Sample descriptions are shown in Table 1.

**Table 1:** Sample descriptive (n = 994).

	India (n = 423)	United Stated (n = 471)
*Gender (%)*		
Male	54.1	45.1
Female	44.6	52.8
Other	0.6	2.1
*Occupational status (%)*		
Full time	56.2	41.5
Part time	10.8	17.2
Unemployed	5.5	15.0
Others (students, retired)	27.5	26.3
*Age*		
Mean	35.2	32.2
SD	11.6	11.9
Max	76	73
Min	18	18
*Age cohorts*		
Gen Z	16.5	30.9
Gen Y	53.9	44.1
Gen X	20.1	17.6
Baby-boomers	9.1	7.2
*Covid infection (%)*		
None	57.1	76.7
Yes (personal or intimate contact)	42.9	23.3

A total sample of N = 994 was recruited, including 473 participants from India and 471 participants from the United States. In India, participants were recruited through Amazon’s Mechanical TurkTM, as well as through social media snowballing. The US sample was recruited via the online platform Prolific. The latter had more female participants, while the Indian sample had more male participants: χ2 (1, N = 929) = 7.05, p = .008. More people in India than in the US reported contracting COVID-19: χ2 (1, N = 945) = 41.02, p < .001. Employment status also differed between the two countries: χ2 (5, N = 910) = 40.94, p < .001, with more full-time workers in India and more part-time and unemployed workers in the US (see also 18 for more information about data collection and sampling).

### 2.2. Measures

#### 2.2.1 Age cohorts

We divided the sample into four age cohorts according to standardized criteria for intergenerational analysis [12, 30]. The first cohort was “Generation Z”, with *n* = 146 (30.9%) US and *n* = 78 (16.5%) Indian participants younger than 25 years. The second cohort, “Generation Y” included *n* = 208 (44.1%) US and *n* = 255 (53.9%) Indian participants aged between 25 and 38 years. The third cohort, “Generation X”, included 83 (17.6%) US and 95 (20.1%) Indian participants aged between 39 and 53 years. The fourth cohort, “baby boomers”, included 34 (7.2%) US and 43 (9.1%) Indian participants older than 54. Compared to the US, in India more participants belonged to Generation X and fewer to Generation Y: χ^2^ (5, *N* = 581) = 37.29, p < .001.

#### 2.2.2 The COVID-19 Multifaceted Threat Scale

The COVID-19 Multifaceted Threat Scale has been developed as a measurement instrument to accurately assess and compare the full range of threat dimensions experienced during the recent pandemic in different cultural contexts. This psychometric tool has been built employing a rigorous scale-validation approach, mixing qualitative bottom-up approaches to item generation with quantitative approaches. This strategy allowed the scale factor structure and evidence of its validity and reliability to be tested across countries that are culturally disparate and differentially impacted by COVID-19. The complete set of procedures and measures used to build this tool are discussed in [18].

For the purposes of the present study, we focus on the “individual threat” sub-scale, which contains fifteen items in total, three items each corresponding to the experience of health (α_US_ = .80; α_India_ = .70), existential (α_US_ = .89; α_India_ = .85), relational (α_US_ = .89; α_India_ = .70), lifestyle (α_US_ = .89; α_India_ = .84) and financial threats (α_US_ = .97; α_India_ = .87). Participants were asked to what extent they felt concerned with regard to the pandemic on a seven-point scale ranging from 1 (*not at all concerned*) to 7 (*extremely concerned*). The items making up the “individual threat” sub-scale of the COVID-19 Multifaced Threat Scale are shown in Table 2.

**Table 2:** The COVID-19 multifaceted threat: Individual sub-scale generative question and items.

Generative question	Please read each of the following statements carefully, and then indicate to what extent you feel worried or concerned with regard to the pandemic situation on a scale ranging from 1 (*not at all concerned*) to 7 (*extremely concerned*).
**Issue 1:** Health Threats	• I might catch COVID-19 • I am very paranoid about germs these days • I am following all the advice to avoid getting sick
**Issue 2:** Existential Threats	• I have a sense of uselessness since the pandemic started • My life has less meaning these days • I feel trapped with no way to escape
**Issue 3:** Relational Threats	• I have had to become less social • I miss my friends • The lack of social contact is noticeable
**Issue 4:** Lifestyle Threats	• I don’t know when my next vacation will be • I do not know when I will be able to travel again • I can no longer visit places outside the area where I live (e.g., seaside, mountains, countryside)
**Issue 5:** Financial Threats	• My financial situation is less stable • I might run out of money • I might not be able to pay my bills

#### 2.2.3 Psychological well-being

Psychological wellbeing was measured using the five-item World Health Organization Well-Being Index [31]. This index averages five statements that respondents rated on a scale from 1 (*At no time*) to 6 (*Every time*) in relation to the previous two weeks: “(…) I have felt calm and relaxed” (α_US_ = .90; α_India_ = .88), “(…) My daily life is filled with things that interest me” (α_US_ = .90; α_India_ = .88), “(…) I have cheerful calm and in good spirits” (α_US_ = .91; α_India_ = .87), “(…) I have felt active and vigorous” (α_US_ = .93; α_India_ = .88), “(…) I woke up feeling fresh and restored” (α_US_ = .90; α_India_ = .85).

#### 2.2.4 Data analysis

Analyses were carried out using SPSS version 28. First, we performed a Multivariate Analysis of Variance (MANOVA) to estimate mean differences on levels of threat experience and psychological well-being between the United States and India. Therefore, a MANOVA was also performed to test possible age effects on psychological well-being and threat scales. Since the scale used to measure age cohorts was a discrete multi-categorical variable, we applied a series of contrasts to identify linear, quadratic and cubic effects. Third, we fitted these contrasts into a mediation model using PROCESS version 3.5 [32] to test whether the age effects on psychological well-being were explained by different threat experiences reported by participants. To reduce the number of estimated parameters, only threat dimensions that differed between generations were included in the mediation model.

## 3. Results

### 3.1. Between-country comparisons in the levels of threat and psychological well-being

Table 3 shows the means, standard deviations and bivariate correlations of all variables of interest for the United States and India separately. In both samples, all threat components correlated positively, suggesting an underlying latent threat factor felt by the participants. As for psychological well-being, both existential and financial threats had negative correlations, while the effects of the threats to health and lifestyle differed between the two countries. The health threat was correlated negatively with psychological well-being in the United States, while the lifestyle threat was correlated negatively with psychological well-being in India.

**Table 3:** Means, standard deviations, and bivariate correlations divided between the USA and India.

	**The US**					
**Variables**	***M (SD)***	**2**	**3**	**4**	**5**	**6**
1 Health threat	4.86 (1.54)	.352***	.320***	.233***	.256***	− .185***
2 Existential threat	3.53 (1.92)		.573***	.384***	.322***	− .298***
3 Relational threat	4.43 (1.79)			.549***	.159***	− .016
4 Lifestyle threat	3.95 (1.84)				.172***	.088
5 Financial threat	3.97 (2.14)					− .191***
6 Psychological well-being	3.46 (1.16)					
	**India**					
**Variables**	***M (SD)***	**2**	**3**	**4**	**5**	**6**
1 Health threat	4.83 (1.29)	.369***	.330***	.308***	.358***	− .071
2 Existential threat	3.78 (1.74)		.411***	.430***	.460***	− .296***
3 Relational threat	4.78 (1.40)			.447***	.274***	− .015
4 Lifestyle threat	4.42 (1.78)				.265***	− .153***
5 Financial threat	4.28 (1.69)					− .169***
6 Psychological well-being	4.17 (1.04)					

***p < .001

Pairwise comparisons extracted from a MANOVA using Pillai’s trace indicated substantial variation in the level of threats and psychological well-being reported by US and Indian participants: *F* (6, 938) = 23.35, *V*_*Pillai*_ = .130, *p* < .001, η^2^ = .130. Pairwise comparisons revealed no differences in the extent of the reported health threat between the two countries: *F* (1, 943) = 0.14, *p* = .706, η^2^ > .001. However, compared to US participants, Indian participants reported more existential threat: *F* (1, 943) = 4.56, *p* = .033, η^2^ = .005; relational threat: *F* (1, 943) = 11.30, *p* < .001, η^2^ = .012; lifestyle threat: *F* (1, 943) = 16.10, *p* < .001, η^2^ = .017; and financial threat: *F* (1, 943) = 6.10, *p* = .014, η^2^ = .006. Compared to US participants (*M* = 3.46, *SD =* 1.55), Indian participants also reported higher levels of psychological well-being: (*M* = 4.17, *SD =* 1.04), *F* (1, 943) = 98.16, *p* < .001, η^2^ = .094.

### 3.2. Age effects on psychological well-being and experiences of threat

**Fig 1:**
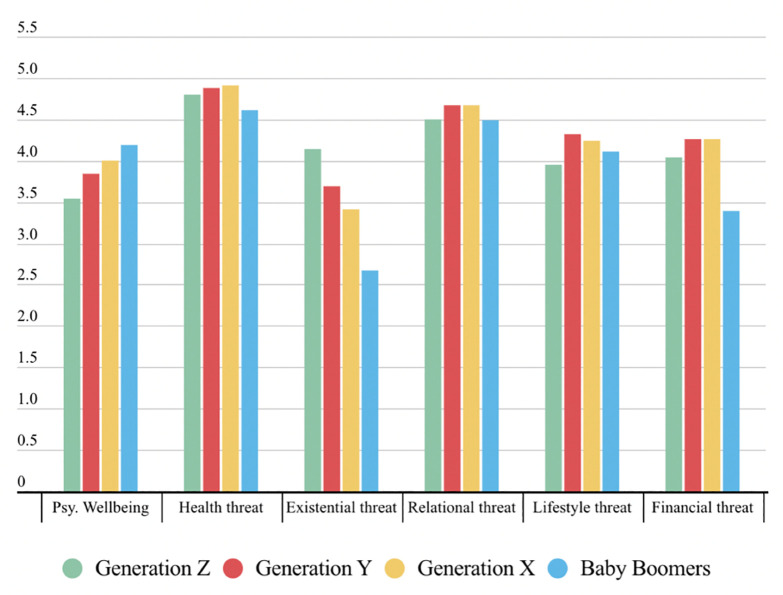
Estimated means and standard errors of the psychological well-being and multiple threat components as a function of generation. Psychological well-being was measured on a six-point scale, threats on a seven-point scale.

To test our hypotheses on psychological well-being and threat experiences in the United States and India, we ran a full-factorial MANOVA, modelling age cohorts as a fixed factor, and threats and psychological well-being as multiple dependent variables. To separate out the country-specific effects, country was included as an additional fixed factor, together with its interaction with age. Furthermore, participants’ gender, and whether they or close others had contracted COVID-19 in the past, were used as statistical controls. Bonferroni correction was applied to reduce the risk of incorrectly rejecting a null hypothesis due to multiple comparisons. Polynomial contrasts were used to capture the possible linear, quadratic and cubic effects of age. Estimated means and standard errors extracted from the analysis are reported in Fig 1.

Multivariate tests revealed a main effect of age cohort: *F* (18, 2742), *V*_*Pillai*_ = .096, *p* < .001, η^2^ = .032, and a main effect of country: *F* (6, 912), *V*_*Pillai*_ = .071, *p* < .001, η^2^ = .071, but not a generation x country interaction: *F* (18, 2742), *V*_*Pillai*_ = .018, *p* = .537, η^2^ = .006. Hence, variations between cohorts and countries in the levels of threats and psychological well-being were independent of each other. In line with H1, the univariate statistics revealed a linear effect of age cohorts on psychological well-being: *b(SE)* = 0.472(.10), *p* < .001, 95% CI [.276; .669], with older participants showing higher levels of well-being (all other polynomial contrasts, *p* > .483). Consistent with a linear effect, pairwise comparisons confirmed a gradual increase in psychological well-being as a function of participants’ cohorts. Only Generation X did not differ statistically from Generation Y (*p* = .552) and Generation Z (*p* = .063). All other group comparisons were significant (*ps* <. 006).

Neither relational nor lifestyle threats varied between the different cohorts. Indeed, none of the polynomial contrasts was significant (all *p*_*s*_ *>*.083). In line with H2, however, the age cohort had a significant linear effect on existential threat: *b(SE)* = − 1.05(.166), *p* < .001; 95% CI [− 1.373, − 0.722], indicating that this decreased with increasing age. Consistent with a linear effect, pairwise comparisons between cohorts confirmed a gradual decrease in existential threats as a function of participants’ ages. Only Generation Z did not differ statistically from Generation Y (*p* = .494). All other group comparisons were significant (*ps* <. 015). Age cohorts also had a significant quadratic effect on financial threat: *b(SE)* = − 0.544(.156), *p* < .001; 95% CI [− 0.850, − 0.238]. This quadratic effect was mainly driven by baby-boomers, who were less concerned by financial concerns compared to all the other cohorts. All other group comparisons were not significant (*ps* >. 067).

### 3.3. COVID-19 multiple threats mediate age effects on psychological well-being

We estimated an indirect effect (i.e. mediation) model using the PROCESS plug-in version 3.5 [32] for SPSS to explore *why* younger cohorts reported lower psychological well-being than older ones. Since we observed significant linear variations between age groups in reported levels of existential threat, we tested whether existential threat could help explain the lower levels of psychological well-being reported by younger participants. Participants’ gender, country and whether they or close others had contracted COVID-19 in the past were used as statistical controls. To capture the linear effects of cohorts on both the mediator and the dependent variable, a linear contrast was used to test our hypotheses. The remaining polynomial contrasts were included as additional controls. As the multivariate analysis of variance showed no Generation X country interaction, country was controlled for and not used as a possible moderator in the mediation analysis. Results are robust and remain consistent when other threat facets are included as additional controls (results upon request).

The results of the mediation analysis are summarized in Table 4. We found (1) a significant total linear effect of age cohorts on psychological well-being: *b(SE)* = 0.110(.022), *p* < .001; 95% CI [0.067, 0.153], meaning that older participants reported 0.11 units higher psychological well-being than younger participants. We also found (2) a significant linear effect of age cohorts on existential threat: *b(SE)* = − 0.236(.037), *p* < .001; 95% CI [− 0.308, − 0.165], indicating that older participants reported 0.24 units less existential threat than younger participants. Finally, we found (3) a significant linear effect of existential threat on psychological well-being: *b(SE)* = − 0.161(.019), *p* < .001; 95% CI [− 0.199, − 0.124], suggesting that with each unit increase in the reported existential threat, well-being decreased for 0.16 units. In line with H3, the linear indirect effect of age cohorts on psychological well-being via existential threat was significant: *b(SE)* = 0.038(.007); 95% CI [0.025, 0.054], as was the residual direct effect of age cohorts on psychological well-being: *b(SE)* = 0.072(.022), *p* = .001; 95% CI [0.029, 0.115].

**Table 4:** Unstandardized effects and standard errors for each path in mediation analysis.

	Existential threat (Mediator)	Psychological wellbeing (Dependent variable)
*Main predictors*		
Age cohorts (linear)	− 0.236 (.037) ***	0.072 (.02) **
Age cohorts (quadratic)	− 0.079 (.072)	− 0.043 (.042)
Age cohorts (cubic)	− 0.028 (.027)	0.006 (.016)
Existential threat		− 0.161 (.019) ***
*Statistical controls*		
Gender female	0.148 (.12)	− 0.158 (.069) *
Indian sample	− 0.303 (.121)*	0.670 (.071) ***
COVID-19 infection	0.269 (.128)*	− 0.014 (.075)

*Note*. Estimates extracted from PROCESS v.3 model 4 inbuilt in SPSS 28. Predictor variables are given in the rows, outcomes variables are given in the columns. The main predictors are reported first, followed by statistical controls

***p < .001

**p < .01

*p < .05, † < .10.

## 4. Discussion

This article shows that younger generations, such as Generation Y and Generation Z, reported lower levels of psychological well-being than older cohorts at the onset of the COVID-19 pandemic. Among the mechanisms associated with these generational inequalities, we observed that higher levels of existential threats significantly accounted for these differences in well-being. Our results corroborate previous research, adding further evidence that the psychological well-being of younger cohorts, while less vulnerable to the physical dangers of the virus, was in no way spared from the side effects of the pandemic [12, 16, 33, 34]. The main contribution of this research is that it yields insights into the mechanisms that contributed to younger peoples’ increased psychological vulnerability. Based on a comparison of different threat dimensions, using the COVID-19 Multifaceted Threat Scale [18, 19], our data suggest that a lack of meaning and purpose, along with perceptions of being “trapped”, weighed particularly on younger cohorts, hence undermining their sense of well-being [20, 21]. While existential threat was the least pronounced threat component overall, being particularly low among “baby-boomers”, its higher prevalence among younger cohorts is alarming. Existential threat means a loss of identity, meaning and the ability to project oneself in the years to come. These are all fears that we know young people experience in the uncertainty typical of the transition to adulthood, but which an event as traumatic as the outbreak of the COVID-19 pandemic may have nurtured and exacerbated [22, 35].

Since our data included participants from India and the United States, we found that these associations were consistent in both samples, corroborating the robustness of our findings. Despite the fact that the two countries implemented similar policies in response to the early stages of the pandemic [27], India and the United States differ significantly in key respects, from cultural norms to socioeconomic structures and in how social inequalities are established in their societies [24, 25]. India’s collectivist culture places a strong emphasis on community ties, and its diverse socioeconomic landscape ranges from urban centres to rural areas. The cultural background of the United States is known to be permeated by values such as individualism, personal fulfilment and autonomy. Despite these key differences, in both samples, this loss of meaning, projection and significance, which in essence represents an existential threat, is associated with the lower levels of psychological well-being among the youngest. This enriches the debate on how the growing precariousness of the youth collective is also inextricably linked to their psychological health, especially in the context of experiencing such difficult periods as the COVID-19 pandemic [36–39].

Previous studies had pointed out that older adults seemed to be more concerned about the economy and their own health at the outbreak of the pandemic, whereas the concerns of younger adults seemed to lie in lifestyle changes and social relationships [12]. We did not observe such significant differences between the cohorts in our analysis, contrary to what was assumed in H2. It emerges more clearly that the baby-boomers felt safer from the financial threat, but this is not associated with their better psychological health. The weight of existential threat as a mechanism for the psychological vulnerability of the younger generations does not offer a causal mechanism, which certainly needs more complex models and data sources. However, what it does offer are indications of how to reflect on this psychological vulnerability of young people. Higher levels of existential threat appear as a response to a “new normal” that constrain their capacity to build the future [17, 40].

On the practical side, the results stressed the demand on policy-makers to pay attention to working with categories such as precariousness and uncertainty, which have been the very essence of youth life for decades [41, 42]. There is a need to develop supportive measures to help young people navigate these uncertainties, now established in youth contemporary life, with their own resources, restoring a sense of action, meaning and purpose. In the wake of disruptive life events, providing platforms for open dialogue and the sharing of experiences could foster a sense of connection and help alleviate and moderate the most sensitive psychological difficulties. Mental health organizations could incorporate resilience-building programmes, equipping young people with the tools they need to cope with change. By addressing the causes of existential threats and developing proactive strategies to manage uncertainty, societies can work to prevent future psychological challenges among young people in the aftermath of crises.

Since our data were collected at the beginning of the pandemic, they cannot inform the development of the models obtained, which is one of the limitations of this article. Our analysis remains part of a cross-sectional study and does not inform the effects these mechanisms may have had over time. However, as the pandemic gripped the world for another two years after the data were collected, it seems almost certain that the psychological burden on younger people has continued [34]. In the younger stages of life, significant events are frequent and often accompanied by major life transitions. At the time the data were collected, Generation Z and Generation Y faced entry into higher education and the labour market and the challenges of early parenthood. It is hard to imagine experiencing these segments of life in the midst of a global pandemic. A further limitation of this study is that it focused exclusively on data collected in the United States and India. As the effects of the pandemic were widespread across the world, the involvement of other countries with different responses to the pandemic, cultural dynamics and economic landscapes could have provided a more complete understanding of the role of existential threats.

## 5. Conclusions

This study has highlighted the pronounced psychological vulnerability experienced by younger generations during the onset of the COVID-19 pandemic. The research underscores how these cohorts exhibited lower levels of psychological well-being compared to older generations. The COVID-19 Multifaceted Threat Scale revealed that existential concerns, encompassing identity, meaning and future prospects, played a pivotal role in shaping their mental health. This finding transcended national boundaries, as participants from both the United States and India experienced similar associations. By shedding light on this specific mechanism, the study urges policy-makers and mental-health practitioners to address the enduring precariousness that defines the lives of young individuals, empowering the younger generation to confront future uncertainties with a renewed sense of purpose. Hopefully, these findings may add a new piece of evidence to the understanding of the psychological vulnerability of young people in the historical contingencies of the COVID-19 pandemic.
